# Cytotoxic effects of auraptene against a human malignant glioblastoma cell line

**Published:** 2019

**Authors:** Amir R. Afshari, Mostafa Karimi Roshan, Mohammad Soukhtanloo, Ahmad Ghorbani, Farzad Rahmani, Mohammad Jalili-Nik, Mohammad Mahdi Vahedi, Azar Hoseini, Hamid R Sadeghnia, Hamid Mollazadeh, Seyed Hadi Mousavi

**Affiliations:** 1 *Department of Pharmacology, Faculty of Medicine, Mashhad University of Medical Sciences, Mashhad, Iran*; 2 *Pharmacological Research Center of Medicinal Plants, Mashhad University of Medical Sciences, Mashhad, Iran*; 3 *Department of Clinical Biochemistry, Faculty of Medicine, Mashhad University of Medical Sciences, Mashhad, Iran*; 4 *Health Promotion Research Center, Zahedan University of Medical Sciences, Zahedan, Iran*; 5 *Department of Physiology and Pharmacology, Faculty of Medicine, North Khorasan University of Medical Sciences, Bojnurd, Iran*; 6 *Medical Toxicology Research Center, Mashhad University of Medical Sciences, Mashhad, Iran*

**Keywords:** Brain tumors, Glioblastoma multiforme, Auraptene, Cytotoxicity, Apoptosis

## Abstract

**Objective:**

Glioblastoma multiforme (GBM) is the deadliest type of primary brain tumors, and the survival of patients is estimated to be only about one year. This study, for the first time, investigated the cytotoxic effects of auraptene on U87 GBM cell line.

**Materials and Methods:**

The cellular toxicity was measured by the MTT assay following 24 and 48-hr treatment with different concentrations of auraptene (0-400μg/ml). Apoptosis was evaluated by sub-G1 peak in cell cycle analysis of propidium-iodide- stained nuclei. Moreover, to determine the Ba*x*, *Bcl-2*, *MCP-1*, *NF-κB*, *IL-1β*, and *p53* genes expression, we used real-time polymerase chain reaction (RT-PCR).

**Results:**

The results revealed that auraptene reduced the viability of U87 cells concentration- and time-dependently with IC_50_ values of 108.9 and 79.17μg/ml obtained for 24 and 48-hr treatments, respectively. Also, sub-G1 population was significantly increased following 24 (p<0.05 and p<0.001) and 48 (p<0.001) hours of treatment. The quantitative real-time RT-PCR showed an up-regulation in *Bax*, *NF-κB*, *IL-1β*, and *p53* but a down-regulation in *MCP-1* and *Bcl-2* genes expression.

**Conclusion:**

This study showed that auraptene triggered apoptosis probably through Bax/Bcl-2 regulation, blocked cell cycle progression and inhibited proliferation in U87 GBM cells. Taken together, auraptene can be utilized as an effective natural medicine against GBM, after complementary studies.

## Introduction

Primary malignant glioma is the most aggressive kind of astrocytic tumor that rapidly proliferates with high local invasion, and the patients only survive about 10-12 months. It is classified into four grades, and the most fatal type is glioblastoma multiforme (GBM), which includes 82% of cases of malignant glioma (Hou et al., 2006 ▶; Omuro and DeAngelis, 2013 ▶). 

The primary treatment for GBM is surgical resection, radiation therapy, and chemotherapy (Afshari et al., 2018 ▶; Yang et al., 2018 ▶). Temozolomide (an oral DNA-alkylating agent) is the standard-of-care chemotherapy, combined with radiotherapy, for GBM (Huang et al., 2017 ▶; Krakstad and Chekenya, 2010 ▶). GBM is characterized by the defect in apoptotic signaling (Bax/Bcl-2 down-regulation) and high proliferation, down-regulation of p53 (as a tumor suppressor gene), and uncontrolled cell cycle (Krakstad and Chekenya, 2010 ▶). 

**Figure 1:**
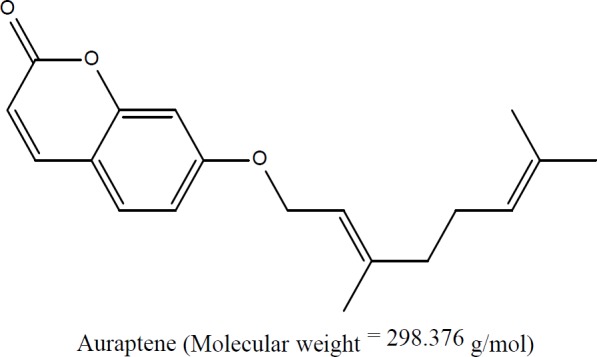
Structure of auraptene

Auraptene (Figure 1) represents the most abundant geranyloxycoumarin found in plants belonging to Rutaceae and Apiaceae families and was seen to possess various promising pharmacological properties (Murakami et al., 1997 ▶; Soltani et al., 2010 ▶; Tanaka et al., 1998 ▶; Tanaka et al., 1997 ▶; Tanaka et al., 2000 ▶). Dietary supplementation of auraptene showed chemo-preventive impacts in animal models of oral, colon, esophagus, liver, breast, prostate, and skin tumors. Modulation of glutathione S- transferase action, decreasing lipid peroxidation, modulation of inflammation, induction of apoptosis, and concealment of superoxide generation have been presented as fundamental components of auraptene chemo-preventive actions (Tanaka et al., 2010 ▶; Tanaka, et al., 1998 ▶). 

Investigation of the mechanisms underlying proliferation and apoptosis offers novel approaches for the treatment of GBM. According to our knowledge, the possible cytotoxic effect of auraptene on GBM has not been studied. Therefore, the present study, for the first time, was designed to investigate the impact of auraptene on proliferation and apoptosis in U87 GBM cell line.

## Materials and Methods

**Cell line and reagents**

The human malignant glioblastoma (U87) cell line was supplied by Pasteur Institute (Tehran, Iran). Auraptene and temozolomide were purchased from Cayman Chemical (Michigan, MI, USA). The 3-(dimethylthiazol-2-yl)-diphenyl tetrazolium (98%, MTT) were obtained from ThermoFisher (ThermoFisher Scientific, Inc.). Trypan blue, propidium iodide (PI), dimethyl sulfoxide (DMSO), penicillin and streptomycin were purchased from Sigma-Aldrich (St. Louis, MO, USA). High Glucose-Dulbecco’s Modified Eagle’s medium (DMEM), fetal bovine serum (FBS), and trypsin-EDTA were purchased from Gibco (Grand Island, NY, USA). 

**Cell culture and treatments**

The U87 cells were maintained in a humidified atmosphere containing 95% air and 5% CO_2_ at 37^°^C. The cells were cultured in high glucose DMEM (4.5g/l) supplemented with 10% v/v FBS and 100unit/ml of penicillin-streptomycin, and the media were changed twice weekly. Then, 12.5mg temazolomide and 25mg auraptene dissolved in 1ml of DMSO. The cells were cultured overnight, the media was changed and treated with different concentrations of temozolomide (0-250μg/ml) and auraptene (0-400μg/ml) for 24 and 48 hours. All the treatments were done in triplicate. 

**Determination of cell viability**

The cell viability was evaluated by MTT assay, which is based on the ability of metabolically active cells to cleave the tetrazolium rings of the yellow MTT dye and form purple formazan crystals (Mosmann, 1983 ▶). In brief, U87 cells (8×10^3^ cells/well) were incubated overnight. Then, the cells were incubated with temozolomide (0-250µg/ml) and auraptene (0-400μg/ml) for 24 and 48 hours. After that, 10μL of the MTT reagent stock (5mg/ml) was added to each well at a final concentration of 0.05% (Hadjzadeh et al., 2006 ▶). After three to four hours, the respected supernatants were removed, and 100μL of DMSO was added to each well to dissolve the formazan crystals. The microplates were shaken gently for 30 min in the dark at 24 °C, and the absorbance was measured at 545 and 630 nm (background) using a Stat FAX303 plate reader. All treatments were carried out in triplicate.

**Cell cycle analysis**

7×10^5^ U87 cells/well were incubated overnight at 37 °C. Culture medium containing the 100 and 400μg/ml of auraptene was added to the medium in each well, and cells were incubated at 37 °C for 24 and 48 hours.

At indicated time points, cells were trypsinized, centrifuged at 2,000 rpm for 5 min at 4 °C, suspended in PBS, and fixed in 70% ethanol at −20 °C overnight. After fixation, the cells were washed and re-suspended in ice-cold PBS. Then, RNase A (100μL) was added to the cells and cells were incubated for 30 min at room temperature. Next, the cells were re-suspended in 400μL PI/Triton-X 100 solution (0.1% Triton-X 100, 0.2mg/ml RNase A and 20μg/ml PI in PBS) for 30 min in the dark. Next, cell cycle distribution was analyzed from 1×10^4^ cells by a BD FACSCALIBUR™ FLOW CYTOMETER (Becton Dickinson, Mountain View, CA, USA). Then, the DNA cell cycle analysis of flow cytometry data was carried out using the software FlowJo ® vX.0.7 (Tree Star, Ashland, OR, USA). All treatments were carried out in triplicate

**Quantitative Real-Time Polymerase Chain Reaction (qRT-PCR)**

Total RNA was extracted from the treated cells (7×10^5^ cells/well, in 6-well plates) using the RNeasy^®^ mini kit (Qiagen GmbH, Hilden, Germany). Quantification and quality control of total RNA was performed in triplicate by a NanoDrop2000 spectrophotometer (Thermo Fisher Scientific, Inc.). Then, RNAs were reverse-transcribed using the Prime-Script ™ RT reagent kit (TaKaRa Holdings, Inc., Kyoto, Japan). Also, quantitative RT-PCR analysis was performed using RealQ Plus 2X MasterMix Green-without Rox™ (Amplicon, Stenhuggervej, Denmark). Next, quantitative RT*-*PCR was performed using specific primers for *Bax*, *Bcl-2*, *p53*, *GAPDH, NF-κB Subunit* *(RELA), IL-1β, and MCP-1 (*Table 1*) which* were obtained from Macrogene (Macrogene Co., Seoul, Korea). The cDNA amplification was performed by using the LightCycler 96 real-time PCR system (Roche Applied Science, Pleasanton, CA, USA). Also, gene expression data were normalized against *GAPDH*. The 2^−ΔΔCt^ method was used to analyze the relative expression of target genes.

The primer sequences (forward and reverse) are listed in Table 1.

**Statistical analysis**

The obtained data were analyzed using software GraphPad Prism ® 6.0 (GraphPad Software, San Diego, CA, USA) and the values were compared by the Tukey’s *post hoc* test. Furthermore, the analysis of cell cycle was done by FlowJo ® vX.0.7 (Tree Star, Ashland, OR, USA) software. A p value less than 0.05 was considered statistically significant. The results are presented as mean±standard error.

**Table 1:** The sequence of primers in the current study

**Gene symbol**	**Gene name**	**Primers (5ʹ → 3ʹ)**	**Accession Number**	**Product length**
*Bax*	*Bcl-2-associated X protein*	*Forward: GGAGCTGCAGAGGATGATTG* *Reverse: CCAGTTGAAGTTGCCGTCAC*	NM_138761.4	*100*
*Bcl-2*	*B-cell lymphoma 2*	*Forward: CTGAGGAGCTTTGTTTCAACCA* *Reverse: TCAAGAAACAAGGTCAAAGGGA*	NM_000633.2	*100*
*P53*	*Tumor suppressor protein*	*Forward: ACCCTTGCTTGCAATAGGTG* *Reverse: AACAAAACACCAGTGCAGGC*	NM_000546.5	*100*
*MCP-1*	*Monocyte chemoattractant protein 1*	*Forward: CATGAAAGTCTCTGCCGCC* *Reverse: GGTGACTGGGGCATTGATTG*	NM_002982.4	*100*
*NF-κB* *Subunit* *(RELA)*	*Nuclear factor-kappa B*	*Forward: GCGAGAGGAGCACAGATACC* *Reverse: CTGATAGCCTGCTCCAGG*	NM_021975.4	*250*
*IL-1β*	*Interleukin-1beta*	*Forward: TGGCAATGAGGATGACTTGTTC* *Reverse: CTGTAGTGGTGGTCGGAGATT*	NM_000576.2	*126*
*GAPDH*	*Glyceraldehyde-3-phosphate dehydrogenase*	*Forward: ACAACTTTGGTATCGTGGAAGG* *Reverse: GCCATCACGCCACAGTTTC*	NM_002046.7	*101*

## Results

**The effects of auraptene and temozolomide on cell proliferation**

**Figure 2:**
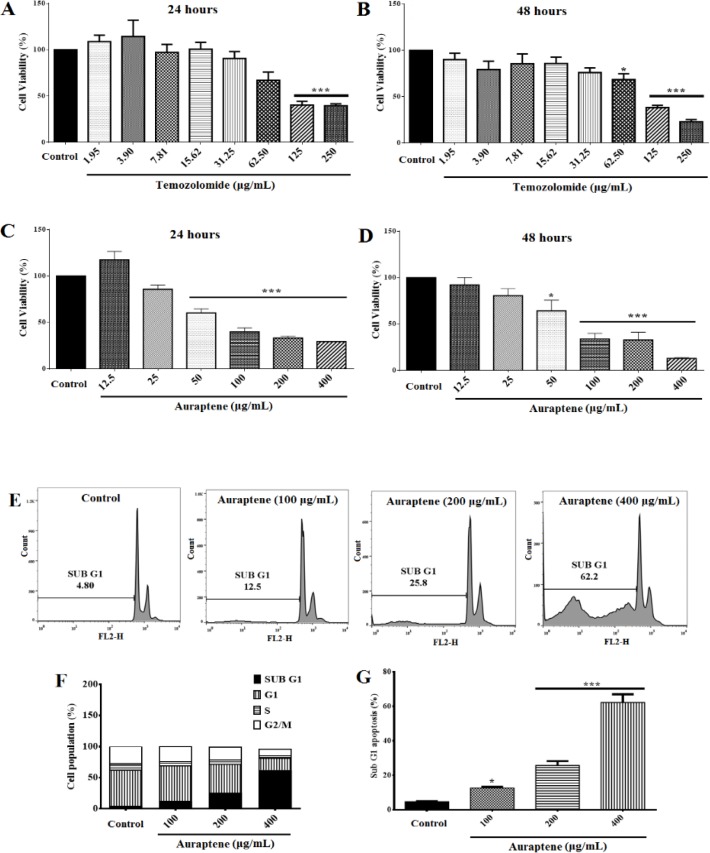
Temozolomide cytotoxicity in U87 cells after 24 (A) and 48 (B) hours. Cell toxicity was evaluated by the MTT assay (mean±standard error). The IC_50_ was 141.7 and 88.42μg/ml for 24- and 48-hr treatment, respectively. (C) and (D) Auraptene cytotoxicity in U87 cells after 24 and 48 hr of treatment, respectively (mean±standard error). Auraptene cytotoxicity was evaluated by the MTT assay. The IC50 was determined at about 108.9 and 79.17μg/ml for 24- and 48-hr treatment, respectively. (E), (F) and (G), Flow cytometry analysis of cell distribution (sub-G1) of U87 cells. Representative histograms of cell cycle distribution depict apoptosis in auraptene-treated (100, 200 and 400μg/ml) U87 cells after 24-hr. (F) The analysis of the cell population at each cell cycle phase relative to total phases. For example, the percent of sub-G1 is measured as the percentage of cells in the sub-G1 phase relative to the number of total cells. Quantification of sub-G1 phase and cell population in each phase were analyzed by FlowJo software (mean±standard error) following 24-hr (G) treatment with auraptene. Each column represents mean±standard error for each group. * p<0.05 and *** p<0.001 show significant differences as compared to the control group (n=8)

To investigate the effects of temozolomide on the viability of U87 cells, we treated the cells with various concentrations (0-250μg/ml) of temozolomide for 24 (Figure 2A) and 48 (Figure 2B) hr and assessed cell proliferation utilizing MTT assay. The results showed that temozolomide significantly decreased cell proliferation 24 (p<0.001) and 48 (p<0.05 and p<0.001) hr after treatment in a concentration- and time-dependent manner. The IC_50_ values, following temozolomide treatment, were about 141.7 and 88.42μg/ml for 24 and 48 hours, respectively. 

Also, The U87 cells were treated with different concentrations of auraptene (0-400μg/ml) for 24 (Figure 2C) and 48 (Figure 2D) hr. Auraptene significantly reduced cell viability by increasing the concentration after 24 (p<0.001) and 48 (p<0.05 and p<0.001) hr. Auraptene IC_50_ values were about 108.9 and 79.17μg/ml for 24 and 48-hr of treatment, respectively. 

**Flow cytometry analysis of cell cycle**

**Figure 3:**
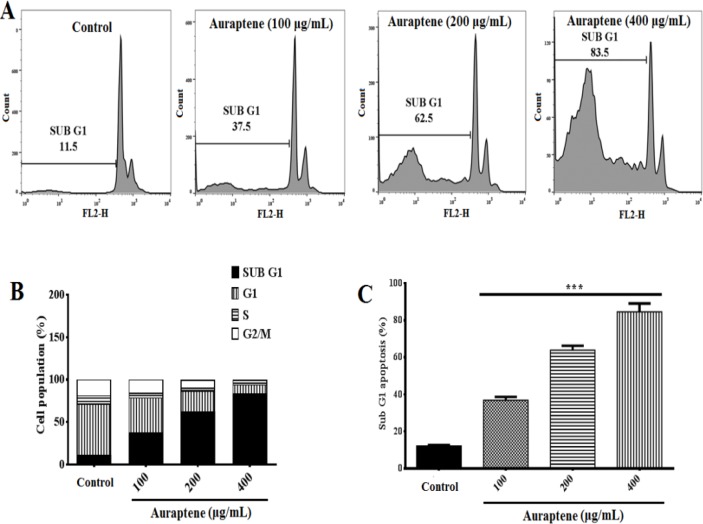
Flow cytometry analysis of cell distribution (sub-G1) of U87 cells. Representative histograms of cell cycle distribution depicting apoptosis in U87 cells treated for 48hr with auraptene (100, 200 and 400μg/ml). (B) Analysis of cell population at each cell cycle phase relative to total phases. For instance, the percent of sub-G1 is measured as the percentage of the number of cells in the sub-G1 population relative to the number of total cells. Quantification of sub-G1 phase and cell population in each phase were analyzed by FlowJo software (mean±standard error) following 48-hr (B and C) treatment with auraptene. *** p<0.001 show significant differences compared to the control group

We examined the effect of auraptene on cell cycle distribution in U87 GBM cell line. U87 GBM cells treated with 100, 200 and 400μg/ml of auraptene for the indicated times (24 and 48-hr), were stained with PI and cell cycle distribution was monitored by flow cytometry. FACS analysis revealed that 24 (Figure 2G, p<0.05 and p<0.001) and 48 (Figure 3C, p<0.001) hours of treatment with auraptene significantly increased the percentage of cells in the sub-G1 phase in a time- and concentration-dependent manner compared to the control group (Figures 2 E, F and G, and Figure 3).

Treatment with auraptene at 100 and 400μg/ml showed a statistically significant increase in sub-G1 phase from 4.8 to 62.2% with a concomitant decrease in S phase from 10.6 to 3.89% following 24-hr treatment (Figure 2E). Also, we observed a remarkable increase in sub-G1 phase from 11.5 to 83.5% with a concomitant decrease in the S phase from 10 to 2.13% following 48-hr treatment (Figure 3A). Figures 2F and G and 3B and C show quantifications of sub G1 phase and cell population in each phase in U87 cells.

**Effect of auraptene on expression levels of the** ***Bax*** **,** ***Bcl-2*** **,** ***p53*** **,** ***MCP-1*** **,** ***NF-κB*** **, and** ***IL-1β*** **genes**

**Figure 4:**
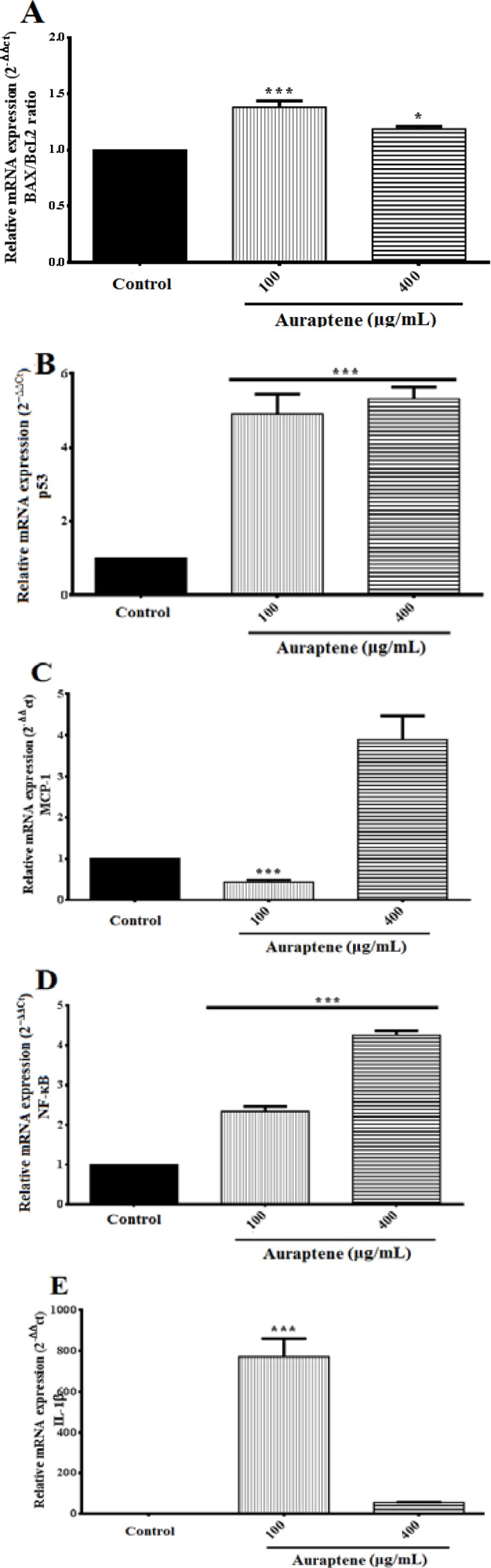
U87 GBM cells were treated with auraptene 100 and 400μg/ml for 24 hr. Total RNA was isolated and mRNA expression was analyzed by quantitative real-time RT-PCR. The relative genes expression levels of *Bax*/*Bcl-2* ratio (A), *p53* (B), *MCP-1* (C), *NF-κB* (D), and *IL-1β* (E) were determined by 2^−ΔΔCt^ method. The y-axis indicates the fold-change. Results were normalized against *GAPDH* in the samples. *p<0.05and ***p<0.001 show significant differences compared to the control group (mean±standard error)

The expression of apoptosis-related genes was evaluated using qRT-PCR. As shown in Figure 4A, we observed an obvious increase in the ratio of Bax to Bcl-2 following treatment with auraptene 100 and 400μg/ml compared to the control group (p<0.001 and p<0.05, respectively). However, the ratio of Bax to Bcl-2 decreased in a concentration-dependent manner. Furthermore, the expression of the *p53* gene, as a tumor suppressor, was remarkably increased in a concentration-dependent manner compared to the control group (Figure 4B, p<0.001). Also, the expression of *MCP-1*, a key chemokine that regulates migration and infiltration of monocytes/macrophages was significantly reduced by auraptene 100μg/ml (Figure 4C, p<0.001), but at the concentration of 400μg/ml, *MCP-1* gene expression was increased compared to the control group. As shown in Figure 4D, *NF-κB Subunit* *(RELA) gene expression was increased* following treatment with auraptene 100 and 400μg/ml compared to the control group (p<0.001). As shown in Figure 4E, *IL-1β*, as a cytokine, was up-regulated following treatment with 100μg/ml of auraptene (p<0.001; the effect of auraptene in 400μg/ml was not significant).

## Discussion

To the best of our knowledge, this study, for the first time, exhibited that auraptene can be a potential anti-GBM candidate. The present study showed that auraptene triggered cell cycle progression and induced cytotoxicity and apoptosis via modulating the expression of transcription genes (*p53*, *Bax*, *Bcl-2*, *MCP-1*, *IL-1β*, and *NF-κB*).

GBM is the most aggressive and deadliest type of astrocytic tumors, with a poor prognosis regardless of the treatment, including tumor surgery, followed by radiation therapy and chemotherapy using the alkylating agent, temozolomide to prevent recurrence (Song et al., 2018 ▶; Tang et al., 2017 ▶). Headways in the previous decades could not improve the general survival rates in patients with this malignant tumor (Awad et al., 2017 ▶; Hilliard et al., 2017 ▶). 

Natural products have been used over the years to prevent and treat many diseases, including cancer as chemotherapy and cytotoxic drugs produce a large number of side effects (Asadbeigi et al., 2014 ▶; Boroushaki et al., 2016 ▶; Jalili-Nik et al., 2018 ▶; Kaur et al., 2018 ▶; Mousavi et al., 2009 ▶; Tavakkol-Afshari et al., 2008 ▶). Medicines obtained from plants are available, inexpensive, safe, and effective, usually have fewer side effects (Afshari et al., 2016 ▶; Boroushaki, et al., 2016 ▶; Mollazadeh et al., 2017 ▶; Sadeghnia et al., 2017 ▶; Shafiee-Nick et al., 2017 ▶), and they are the source of many potent clinical anti-cancer molecules (Shafiee-Nick, et al., 2017 ▶; Solowey et al., 2014 ▶). 

In the present study, we sought to determine the cytotoxic effects of auraptene on the U87 GBM cell line. Some studies showed the potential inhibitory effects of other natural and chemical compounds on the GBM cell line (Lefranc et al., 2009 ▶; Liu et al., 2013 ▶). This is the first study to examine the cytotoxic effect of auraptene on the U87 GBM cell line. 

Previous studies on auraptene demonstrated its anti-tumor effect on other cell lines (Jun et al., 2007 ▶; Kohno et al., 2006 ▶; Tanaka, et al., 2000 ▶). In a study done in 1996, auraptene, as an active flavonoid compound derived from *Citrus aurantium *L. showed a cell–growth inhibitory effect against human myelogenous leukemia and mouse lymphocytic leukemia cells, *in vitro* (Gholami and Shamsara, 2016 ▶). Auraptene was shown to suppress gastrointestinal malignancies as well (Takeda et al., 2007 ▶). The dietary administration of auraptene fundamentally repressed 4-nitroquinoline 1-oxide -induced tongue tumorigenesis in conjunction with decreased recurrence of dysplastic lesions, the expression of the cell proliferation biomarker, induction of phase II enzymes glutathione S-transferase, and quinone reductase in the liver and tongue (Tanaka et al., 1998 ▶). It was also shown that auraptene suppressed cell proliferation in the esophageal epithelium and inhibited tumor development induced by N-nitroso methyl benzylamine (Kawabata et al., 2000 ▶).

In the present study, it is shown that cell proliferation was influenced by various concentrations of auraptene (0-400μg/ml, Figures 2C and D), depending on the time and concentration. Also, 50% inhibition of cell proliferation (IC_50_) values of 108.9 and 79.17μg/ml, following 24 and 48-hr treatment were observed, respectively. However, the IC_50_ of temozolomide, as an approved drug for GBM, was 141.7μg/ml and 88.42μg/ml in 24- and 48-hr treatments, respectively (Figures 2A and 2B). The IC_50_ of auraptene in GBM cells was lower than that of temozolomide after 24-hr of treatment. Hence, the results of this study suggest that auraptene has higher cytotoxicity than temozolomide, which is the preferred drug for the treatment of GBM. It was also observed that the IC_50_ of auraptene decreases after 48-hr treatment. Hence, it can be concluded that the cytotoxic effects of auraptene on cell proliferation increase in a time-dependent manner. 

The molecular investigations of human cancers revealed that cell cycle regulators, including transcription activator and cyclin-dependent kinase (CDK) regulators, are frequently mutated in many types of malignancies (Graña and Reddy, 1995 ▶; Kastan et al., 1995 ▶; Mollazadeh, et al., 2017 ▶). Critically, control of cell cycle progression in cancer cells is thought to be a conceivably successful approach for controlling tumor growth and development (Liu et al., 2016 ▶). Natural products have been reported to arrest the cell cycle at G0/G1, S or G2/M stage (Gamet-Payrastre et al., 2000 ▶; Li et al., 2007 ▶; Massagué, 2004 ▶). Our study showed that, auraptene (100, 200, and 400μg/ml) significantly increases the sub-G1 peak following 24- (Figures 2E, F, and G) and 48-hr (Figure 3A, 3B, and 3C) treatment, which suggests that auraptene can stop the progression of cell cycle through induction of apoptosis. 

Inflammation in GBM includes invasion of hematopoietic cells, tissue edema, and release of cytokines (Ha et al., 2014 ▶). Monocyte chemoattractant protein-1 (MCP-1) is a chemoattractant cytokine for monocytes and macrophages in the process of inflammation (Leonard and Yoshimura, 1990 ▶). Higher level of MCP-1 expression seen in GBM compared to the lower grade of glioma, and expression of MCP-1 by tumor cells is believed to stimulate the recruitment of macrophages that could actuate angiogenesis and edema, two histopathological highlights of GBM (Desbaillets et al., 1994 ▶; Platten et al., 2003 ▶; Salcedo et al., 2000 ▶). Since *in vitro*-cultured tumor cells often produce significant amounts of MCP-1, tumor cells are considered the main source of MCP-1 (Hambardzumyan et al., 2016 ▶; Park et al., 2018 ▶). This study showed that auraptene at the IC_50_ concentration could diminish *MCP-1* gene expression (Figure 4C), which mediates anti-inflammatory effects. 

Furthermore, as cells become old or damaged, they undergo apoptosis, necrosis or both and are supplanted with new cells (Moradzadeh et al., 2017 ▶; Vazifedan et al., 2017 ▶). Chemotherapy kills tumor cells through induction of apoptosis and necrosis (Furnari et al., 2007 ▶; Hao et al., 2001 ▶). In apoptosis, an imbalance between proteins Bax and Bcl-2 could be found (Bukhari et al., 2018 ▶; Kaufmann and Earnshaw, 2000 ▶; Lowe and Lin, 2000 ▶). Auraptene, in the present study, triggered apoptosis in cancer cells at concentrations of 100 and 400µg/ml, by increasing Bax/Bcl-2 ratio (Figure 4A). 

Moreover, p53 (as a tumor suppressor protein) is the most commonly mutated pathway in tumorigenesis (Harris et al., 2018 ▶) and a crucial component in multicellular organisms, as it regulates the cell cycle progression, tumor growth and development, tumor invasion, and helps to prevent cancer via induction of apoptosis (i.e. through Bax/Bcl-2 regulation) (Aylon and Oren, 2017 ▶; Ferraz da Costa et al., 2017 ▶). In the past decade, activation of the p53 growth inhibitory pathway in GBM has been seriously investigated to suppress GBM progression (Cerrato et al., 2004 ▶; Naumann et al., 2001 ▶). Mutations in the p53 pathway are detected in GBM and cause instability in the GBM tumor microenvironment (Lee et al., 2012 ▶; Wang et al., 2013 ▶). The present study showed that auraptene increased the gene expression of *p53* at mRNA level in U87 GBM cells, which could subsequently activate apoptosis and disrupt the cell cycle progression (Figure 4B).

The nuclear factor (NF)-κB proteins are a group of transcription factors that regulate lots of genes associated with cell growth, development, differentiation, and apoptosis (Dolcet et al., 2005 ▶). Different studies showed that activation of NF-κB promotes p53- mediated apoptosis (Ryan et al., 2000 ▶) and the activated NF-κB is a predictor of good prognosis in gastric cancer (Lee et al., 2005 ▶). Also, Hogerlinden et al. showed that inhibition of NF-κB might be effective against squamous cell carcinomas (van Hogerlinden et al., 1999 ▶). The present study revealed that auraptene is able to up-regulate *NF-κB* (Figure 4D) and *p53* genes expression that may be due to its increasing effect on *IL-1β* gene expression (Figure 4E) that prompts oxidative stress and cell death. 

Taken together, this study is the first work reporting that auraptene exerts cytotoxic effect against U87 GBM cell line. Our findings suggest that auraptene inhibits proliferation of U87 GBM cells through blocking cell cycle and modulating transcription genes, implying that auraptene may be a useful natural chemotherapeutic agent for future GBM treatment. 
